# Advancing clinical reasoning in virtual patients – development and application of a conceptual framework

**DOI:** 10.3205/zma001159

**Published:** 2018-02-15

**Authors:** Inga Hege, Andrzej A. Kononowicz, Norman B. Berman, Benedikt Lenzer, Jan Kiesewetter

**Affiliations:** 1 LMU Munich, Institute for Medical Educaiton, Munich, Germany; 2 Jagiellonian University Medical College, Department of Bioinformatics and Telemedicine, Krakow, Poland; 3 Geisel School of Medicine, Dartmouth, USA

**Keywords:** Virtual Patients, Clinical Reasoning, Qualitative Research, Healthcare Education

## Abstract

**Background: **Clinical reasoning is a complex skill students have to acquire during their education. For educators it is difficult to explain their reasoning to students, because it is partly an automatic and unconscious process. Virtual Patients (VPs) are used to support the acquisition of clinical reasoning skills in healthcare education. However, until now it remains unclear which features or settings of VPs optimally foster clinical reasoning. Therefore, our aims were to identify key concepts of the clinical reasoning process in a qualitative approach and draw conclusions on how each concept can be enhanced to advance the learning of clinical reasoning with virtual patients.

**Methods: **We chose a grounded theory approach to identify key categories and concepts of learning clinical reasoning and develop a framework. Throughout this process, the emerging codes were discussed with a panel of interdisciplinary experts. In a second step we applied the framework to virtual patients.

**Results: **Based on the data we identified the core category as the "multifactorial nature of learning clinical reasoning". This category is reflected in the following five main categories: Psychological Theories, Patient-centeredness, Context, Learner-centeredness, and Teaching/Assessment. Each category encompasses between four and six related concepts.

**Conclusions: **With our approach we were able to elaborate how key categories and concepts of clinical reasoning can be applied to virtual patients. This includes aspects such as allowing learners to access a large number of VPs with adaptable levels of complexity and feedback or emphasizing dual processing, errors, and uncertainty.

## Zusammenfassung

**Hintergrund:** Klinisches Entscheiden ist eine komplexe Fähigkeit die Medizinstudierende während ihrer Ausbildung erwerben müssen. DozentInnen fällt es dagegen oft schwer, ihren Entscheidungsprozess Studierenden zu erklären, da es dieser Prozess oft unbewusst abläuft. Der Einsatz von virtuellen Patienten (VPs) ist sinnvoll, um das Erlernen von klinischem Denken zu unterstützen. Es ist allerdings bis heute nicht ausreichend erforscht, welche Eigenschaften bzw. Rahmenbedingungen das Erlernen von klinischem Entscheiden am effektivsten fördern. Unser Ziel war es zunächst Schlüssel-Konzepte des klinischen Entscheidungsprozesses mit einem qualitativen Ansatz zu identifizieren. In einem zweiten Schritt haben wir Schlussfolgerungen gezogen, wie die einzelnen Konzepte weiterentwickelt werden können, um das Erlernen von klinischem Entscheiden mit virtuellen Patienten zu verbessern.

**Methoden: **Mittels grounded theory wurden Hauptkategorien und -konzepte des Erlernens von klinischem Entscheiden identifiziert und ein Framework entwickelt. Im Laufe dieses Prozesses wurden die sich entwickelnden Codes mit einer interdisziplinären Expertengruppe diskutiert und in einem zweiten Schritt das Framework auf virtuelle Patienten angewendet.

**Ergebnisse: **Aus den Daten entwickelte sich als Kernkategorie die „multifaktorielle Natur des Erlernens von klinischem Entscheiden“. Diese Kernkategorie spiegelt sich in den folgenden fünf Hauptkategorien wieder: Psychologische Theorien, Patientenorientierung, Kontext, Lernerzentrierung und Unterrichten/Prüfen. Jede dieser Kategorien umfasst wiederum vier bis sechs zugehörige Konzepte.

**Schlussfolgerungen: **Mit unserem Ansatz haben wir herausgearbeitet wie die einzelnen Konzepte des klinischen Entscheidens auf virtuelle Patienten übertragen werden können. Dies beinhaltet Aspekte wie den Lernenden das Üben mit vielen VPs zu ermöglichen oder VPs mit anpassbarer Komplexität und Feedback bereitzustellen. Darüberhinaus sollten Aspekte wie dual processing, Fehler und Unsicherheit berücksichtigt werden.

## Background

Clinical reasoning and related concepts such as clinical decision making or problem-solving have been major topics of healthcare education research. Clinical reasoning is a complex set of skills that encompasses the application of knowledge to collect and integrate information from various sources to arrive at a (working) diagnosis and management plan. The symptoms and findings of a patient have to be matched to a set of differential diagnoses to be able to arrive at a working or final diagnosis. Although clinical reasoning is a fundamental skill and one that has been the focus of research for over 30 years, it is not yet fully understood [1], [2] . 

Healthcare students must acquire clinical reasoning skills and continue to build upon them in their clinical work [1]. However, experienced healthcare instructors often find it difficult to slow down and fully explain their clinical reasoning; reasons include the dynamic and often unconscious components of the reasoning process [1] and a lack of formal training [2]. 

During medical school clinical reasoning is often taught in bedside teaching courses or problem-based tutorials, but virtual patients are becoming a more important teaching activity to train and assess this skill to prepare students for real patient encounters [3], [4].

VPs in healthcare education are interactive computer-based programs that simulate real-life clinical scenarios [5]. The form of VPs varies greatly and ranges from basic text-based scenario descriptions to high-fidelity software simulations or virtual reality scenarios [6]. Evidence suggests their use in the form of interactive patient scenarios supports student clinical reasoning skills [7]. The first articles about using computers to simulate clinical reasoning for students were published in the 1970s [8] and VPs have gained popularity in healthcare curricula over the past 40 years. VPs have been integrated into undergraduate healthcare curricula in different formats, such as blended-learning scenarios [9], during clinical clerkships [10], or as assessment tools [11].

The knowledge gap this study intends to address was recognized years ago and remains largely unaddressed. Cook at al. suggested that VPs should be ideally suited to teach clinical reasoning, but that how learning occurs is not yet fully understood. Further, they concluded that there is insufficient evidence how specific VP design variations, such as content, authenticity, interactivity, or feedback, support clinical reasoning acquisition [7]. How VPs model and influence clinical reasoning and consequently, how VPs should be presented to healthcare students remain unanswered questions. 

To advance this question, we decided to "zoom out" from VPs and VP technology as elaborated by Edelbring et al. [12] Thus, our aims were

1. to identify key concepts of learning clinical reasoning in general and
2. evaluate how these concepts are reflected in virtual patients to
3. elaborate recommendations on how to advance the learning of clinical reasoning with virtual patients.

## Methods

**Figure 1:**
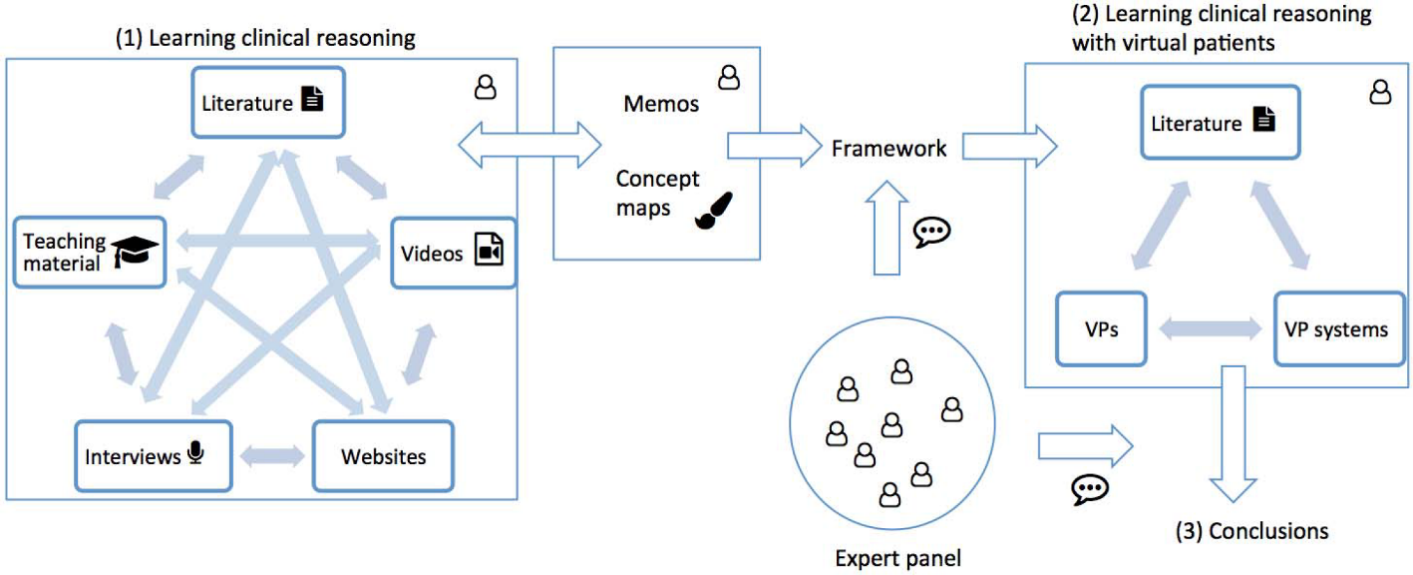
Study design with two steps: Exploration of learning clinical reasoning with framework development based on memos and concept maps, and application of the framework to virtual patients and elaboration of conclusions.

We chose a qualitative approach based on Glaserian grounded theory [13] to explore the broad topic of clinical reasoning skills acquisition. Grounded theory is an inductive research methodology to understand a phenomenon and develop a theory which is anchored in the data. In an iterative process data collection, coding, and the development of the theory interact with each other [14]. Thus, grounded theory aligned with the focus of our research and allowed us to explore and include a wide range of data sources into our analysis. Figure 1 (Fig. 1) shows an overview of the study design. Following the grounded theory dictum "all is data" [13] we included a wide range of data sources, such as scientific literature, videos, or websites, into our analysis, since our approach was focused on synthesizing descriptions, frameworks, and teaching approaches of clinical reasoning. In a second step we explored how the emerged framework is represented in virtual patients and introduced conclusions on how to improve virtual patients accordingly. 

One researcher (IH), an experienced healthcare education researcher, conducted the study in close communication and cooperation with all authors.

### Data collection and analysis

The main researcher started the grounded theory with the book "Developing clinical problem-solving skills" by Barrows et al. [15], which was coded completely. The book covers a broad range of teaching clinical reasoning aspects, and therefore we found it to be an ideal starting point for the analysis. In a cyclic process [16] the researcher applied theoretical sampling based on memoing to guide further data collection. Based on emerging codes she searched for data sources that could

1. further explain,
2. contrast, or
3. add a new perspective.

The Google search engine and PubMed were used as search tools for data sources, additionally, references of a data source were considered as potential new data sources. The search was not limited to a particularly time frame or source or authors.

Before coding a data source the researcher briefly scanned the abstract or summary and decided about its inclusions if at least one of the above described criteria was fulfilled.

For example, during coding the book by Barrows et al. the theme "role of the teacher" emerged, which required further elaboration. The researcher then identified and explored other sources such as an article by Eva [17] or the Massive Open Online Course (MOOC) of the University of Michigan about Instructional Methods in Health Professions Education [https://www.coursera.org/learn/instructional-methods-education] to further elaborate this theme. 

If the emerging themes could not sufficiently be explored with articles or online resources, the researcher conducted interviews with different stakeholders, such as healthcare educators or researchers to further explore the themes. For example, "learning from errors" and "concept mapping in clinical reasoning" were further explored in interviews (see Attachment 1 for details about all conducted interviews). The interviews were colloquial conversations recorded in field notes; they were held online or face-to-face in a private setting.

A process schema with all data sources and why and when in the process they were included is available on request. 

Before the core category - a core theme that integrates all lower level categories - emerged, the researcher open-coded each data resource; after that she applied selective coding to elaborate relations between concepts and further specify the nature of the categories. Codes were constantly compared, revised, and merged into concepts. During the process, the researcher composed concept maps and memos, which included the date of analysis, a brief summary, relations of key aspects, suggestions for further themes to explore, emerging ideas, and specific links to the data source to document the analysis. 

Overall, 107 data sources, including six interviews, were coded; a full list can be found in Attachment 1.

Consistent with a theoretical sampling approach, the researcher continued the data collection until saturation was reached, i.e. no additional data were found to develop new themes in categories or new relationships between the themes. The memos and concept maps were then analyzed and a framework constructed based on the emerging categories, concepts, and relations. 

#### Evaluation of virtual patients based on the framework

In a second step we transferred the framework to the virtual patient context and analyzed resources, such as VP-related literature, virtual patients and VP systems (a full list of data sources can be found in Attachment 2). The researcher analyzed virtual patients and authoring guidelines [http://www.virtualpatient.eu], [http://vpsystems.virtualpatients.net] based on the categories and concepts of the framework. The findings were complemented with an analysis of scientific articles which were extracted in a purposive literature search for each category.

The researcher then explored how concepts were implemented in these VPs and, similar to the grounded theory approach, documented the findings in memos and concept maps. 

We discussed the developed concepts, theoretical saturation, the emerging framework, and our conclusions on virtual patients within an interdisciplinary panel of two healthcare educators, two healthcare professionals , two healthcare education researchers, an undergraduate medical student, and a computer scientist. The concept maps and memos served as a basis for these discussions. 

#### Ethical approval

We received ethical approval from the Ethical Committee of the University of Munich for the interviews conducted for this study. 

## Results

**Figure 2:**
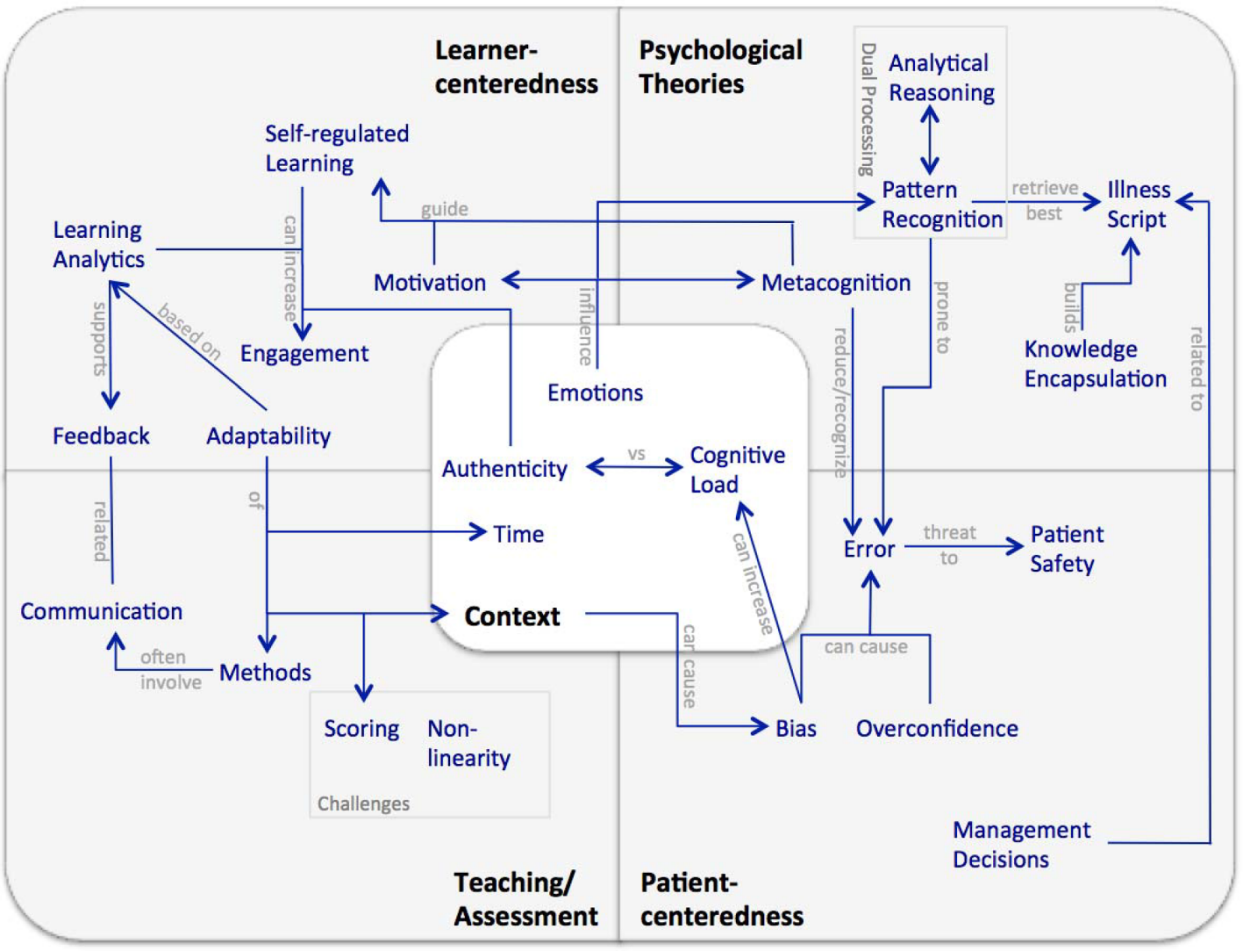
Framework of learning clinical reasoning with the five main categories, related concepts, and interrelations derived in the first step of the study.

During the grounded theory analysis we identified the core category "multifactorial nature of learning clinical reasoning". Based on the data we elaborated the following five categories with main actors: Learner-centeredness (learner), Patient-centeredness (patient), Psychological Theories (researcher), Teaching/Assessment (teacher), and Context (healthcare professional). Categories and their dedicated concepts are connected to the core category, but also interrelated with each other (see Figure 2 (Fig. 2)). 

Below, we will elaborate on the results of each category, give examples on how they are grounded in the data, and introduce findings from the analysis of the virtual patient presentations based on the framework. A summary of the results is shown in Attachment 3. Detailed concept maps for each category and an overall map showing the relations of the data sources and the concepts can be provided on request. 

### Context

Context emerged as an overarching concept that influences most aspects of clinical reasoning and learning [18]. In a clinical encounter patients and healthcare professionals interact in a certain environment, or context, with each other. Emotions influence this interaction and are an important component of the clinical reasoning process [19]. The learning context - where, with whom, and how the learning takes place - is also relevant [20]. In such a learning environment the balance between authenticity and cognitive load influences the learning experience [21], [22].

Time is an important aspect in different ways. First, it plays a role in a clinical encounter in terms of time pressure or when an encounter takes place - for example late at night or during the day. Secondly, learners need time to develop clinical reasoning skills [23], but, in learning contexts time may be restricted due to curricular constraints. And thirdly, time can be an indicator for efficient clinical reasoning (pattern recognition) or a deliberate analytical process.

In contrast to a real patient encounter the learning and the clinical context are separated in a VP environment. Time influences the clinical setting as well as the learning environment [24]. However, VPs are typically static – learners are either rewarded for a long and deliberate engagement with a VP or for a quick and time-efficient problem solving approach.

#### Learner-centeredness 

Learner-centeredness emerged as an important category for the learning of clinical reasoning [2]. The concepts in this category are tightly interrelated with each other and other categories (see Figure 2 (Fig. 2)). In self-regulated learning (SRL) environments the learner is autonomous and controls his own learning activities and settings, which can increase his engagement and motivation [25]. Deliberate and active engagement is necessary to learn clinical reasoning skills [26]. Adaptability emerged as dynamically adjusting learning content, context [18], feedback [27], scaffolding [28], or other activities to the learners' needs and level of expertise [29]. For example, feedback can be adaptable in terms of who provides feedback – an instructor, peers, or the VP – and when, or how detailed it is given. 

Learning analytics – i.e. the collection and analysis of learner activities – can form the basis for adapting a learning activity based on the learner's skill level in clinical reasoning. Also, learning analytics can be the basis of the feedback to the learner and recommend next activities [30]. 

Virtual patients are learner-centered, adaptable activities [31], [32] however, the degree of learner-centeredness is limited, because VPs are often used to standardize teaching and assessment [33]. 

#### Psychological Theories

This category is based on psychological theories and frameworks that have been developed to explain the clinical reasoning process. It includes dual processing, which encompasses analytic reasoning, pattern recognition (i.e. non-analytic reasoning), and a continuum between these two approaches [34]. Also, the concept of knowledge encapsulation, a process that supports the development of illness scripts [35] emerged in this category. Both, illness scripts and knowledge encapsulation are prerequisites for pattern recognition and are more prominent in experts than in novice learners. Another concept is metacognition [e.g. 25] - thinking or reflection about one's own thinking. It is an important strategy to reduce or process cognitive errors in clinical reasoning [36]. Evidence suggests that for developing expertise novices have to compare and contrast many patient cases, to encapsulate their knowledge and develop individual illness scripts [37]. 

In addition to seeing real patients at the workplace, learning with standardized patients or paper-based cases, virtual patients can be an answer to this requirement, for example by allowing deliberate practice. 

To support analytical reasoning most VPs implicitly include components of an illness script, such as a problem list, differential diagnoses or a final diagnosis [38], which can also form the basis for authoring a VP scenario. Learners are either prompted to construct these components or they are automatically revealed in the scenario. 

#### Teaching/Assessment

Assessment and teaching emerged as strongly interrelated with learning of clinical reasoning, but this category focuses more on the educator's view on what and how a learner should learn and be assessed. 

Because clinical reasoning is a complex and non-linear process, evaluating and scoring learning or assessment activities is challenging [39]. Often there is no single correct answer and uncertainty and ambiguity need to be addressed. 

Numerous methods to assess different steps in the clinical reasoning process have been described [40]; few, such as script concordance testing [41] or concept mapping [42] reflect the non-linear aspects of clinical reasoning. 

Based on the data sources, we coded communication as a teaching/assessment concept, that is mainly related to teaching and assessment of clinical reasoning skills [43], although it is an important aspect for all interactions between actors. Typically, assessment of clinical reasoning skills at the workplace involves communication between a learner and an instructor or senior physician; for example, a learner presenting a summary statement of a patient or elaborating about possible differential diagnoses [43]. 

VPs are used as assessment tools, especially for formative assessments [44], and they also include a variety of methods to assess clinical reasoning skills, such as multiple choice questions or decision points. However, the non-linearity of clinical reasoning is a challenge for scoring and feedback and the quantitative methods often implemented in VPs, do not sufficiently reflect this. 

#### Patient-centeredness

This category encompasses concepts related to patients, such as patient safety and errors, which may not be covered sufficiently in healthcare education [45]. Different types of errors, such as premature closure, related to different steps in the clinical reasoning process, may occur and consequently affect the patient [46]. Biases (e.g. gender or confirmation bias) or overconfidence with one's own decisions can cause such cognitive errors [36]. 

Another concept in this category is management decisions. Although related to illness scripts, the management of patients with the same disease is heterogeneous and management decisions need to be individually tailored to the patients' needs [18].

Virtual patients provide a safe environment for learners, patients and educators, which is crucial to allow learners to make errors and to learn from them without threatening a patients' safety [https://members.aamc.org/eweb/upload/Effective%20Use%20of%20Educational.pdf], [47]. 

The patient presentation in a VP varies from one-sentence descriptions, which neither address the patient by name nor give him a face, to more patient-centered representations [48], which include media of the patient and his/her reactions and emotions. 

Attachment 3 summarizes the results of the second step of the qualitative exploration, in which we applied the elaborated framework to the state-of-the-art research and development of virtual patients. The Attachment 3 also includes open questions, which should be kept in mind when designing or providing virtual patient-based learning scenarios. 

## Discussion

We identified five categories and related concepts for "learning clinical reasoning" in a grounded theory approach. The developed framework (see Figure 2 (Fig. 2)) visualizes the main components and interrelations we have identified in our study. It is fairly complex, but we believe it adequately reflects the core category "multifactorial nature of learning clinical reasoning" and the diversity and richness of the topic. 

Based on the five categories and concepts of the framework we suggest improvements in the design and use of VPs for learning clinical reasoning.

### Context

The separation of the learning and clinical context in VP scenarios offers the opportunity to let the learners adjust both contexts separately to their needs. We suggest providing an individually increasingly challenging context of the virtual clinical encounter, for example by including irrelevant information or emotionally complex situations depending on the learner's skill level and goals. However, we consider it important to balance authenticity and cognitive load to not overburden the learners.

The often static nature of VPs does not reflect the dynamic character of clinical reasoning. Instead of considering the time on task as a fixed scoring component we suggest including it as component to adapt VP complexity. For example, the available time of the virtual clinical encounter could be limited (time-pressure) for more advanced learners or those who want to challenge themselves. To simulate a clinically authentic and time-pressured environment the learner can also be challenged to solve multiple VPs simultaneously.

#### Learner-centeredness

We propose a more flexible environment to allow learners to deliberately practice individually or in a team. Learners should be able to choose the level and type of feedback or adapt aspects such as context and content complexity. Computational models integrated in virtual patients can be the basis for such adaptable content

A VP system may recommend next activities, not necessarily limited to VPs, or further adaptions based on the analysis of learner's performance, level of self-direction, and self-defined learning goals for each step of the clinical reasoning process. Such an approach would enable learners to focus on individual areas of weakness. Continuous, timely, and specific feedback should be provided by the VP system, an instructor, or in form of summative peer or expert responses. 

#### Psychological Theories

Although there are still many open questions concerning the exact nature of dual processing, there is evidence for a continuum or interaction between the process of pattern recognition and analytical reasoning. We envision a dynamic representation of the clinical reasoning process in VPs that allows learners to apply analytical reasoning, pattern recognition, or some combination thereof depending on the complexity of a VP, their skill level and goals. 

We recommend creating a large pool of short and focused VPs to cover a variety of problems and allow learners to select VPs of varying clinical presentations and levels of complexity.

We recommend uncovering the implicit link between VPs and the illness scripts they are based on. This would engage the learner in actively and explicitly building their own illness scripts based on the VP scenarios. Such individually created illness scripts form stand-alone learning achievements, and learners may be encouraged to further develop and enrich them even outside a VP environment. To support pattern recognition learners could also be allowed or even encouraged to skip steps of the illness script building and make a diagnostic or management decision at any stage of the VP scenario.

#### Teaching/Assessment

To better reflect the non-linear character of clinical reasoning in VPs, we suggest assessing clinical reasoning as a multi-step process [3] using a variety of assessment and feedback methods for each step, including qualitative methods. For example, concept mapping could be an ideal method to visualize the VPs illness script. Consequently, that requires applying more complex scoring algorithms that take into account the diversity of such maps.

#### Patient-centeredness

A minimalistic patient presentation reduces the cognitive load for the learner, but does not adequately represent the patient in the clinical reasoning process. Also, it does not account for other factors that influence the clinical reasoning process, such as emotions, communication challenges, or biases. 

On the other hand, media-rich VPs are more time-consuming for learners to work through, thus, contradicting the concept of working through many cases to develop illness scripts. To balance these two approaches, we recommend providing at least a basic description of the patient (including name, age, and some contextual information) and an image of the patient in all VPs and enrich some with additional media and elaborated descriptions to more adequately address the patients' role and emotional situations.

Virtual patients provide learners a safe environment in which they can learn at their own pace without harming a real patient. Therefore, we suggest including errors into VP scenarios and we envision advancing this concept by provoking errors and explicitly including potential causes, such as biases. In addition, immediate feedback, an elaboration of the error, and strategies for avoidance should be provided. 

Finally, we suggest more explicitly covering the individual nature of management decisions, which can vary from patient to patient. Factors influencing these decisions should be elaborated.

#### Limitations 

We are aware that our study has several limitations. Due to the large amount of data resources, especially literature, related to clinical reasoning, decision making and problem solving, our data sample size was relatively small, even if care was taken to ensure broad and covering sampling and theoretical saturation was discussed with an interdisciplinary panel of content matter experts.

We are also aware that the study design is unusual, since we did not discuss the emerging framework directly, but its application to virtual patients. 

Despite our effort to form an interdisciplinary panel of experts representing a wide range of perspectives, we were unable to include patients.. However, patients are important stakeholders in the process of learning clinical reasoning and we suggest further research to include their view. 

Except for the lack of patient's perspective we found the size and diversity of the expert panel (seven members) ideal for discussing the study. However, we cannot exclude that with a larger number of experts new perspectives might have arisen and influenced the final framework.

## Conclusions

In our two-step approach and "zooming out" from virtual patients we developed a framework for learning clinical reasoning. In the second step we applied this framework to the world of virtual patients and drew conclusions on how clinical reasoning in VPs can be enhanced to be more effective learning resources. 

Based on these conclusions we will develop a platform-independent open source clinical reasoning toolbox that can be integrated into VP scenarios. Furthermore, we plan to implement a large-scale, cross-institutional study to evaluate our approach. The results of this study will inform the further development of clinical reasoning in VPs. 

Due to the general character of the framework, we believe that it is also applicable when developing or advancing clinical reasoning curricula or faculty development courses about clinical reasoning. 

## Funding

This project (IH) receives funding from the European Union’s Horizon 2020 research and innovation programme under the Marie Sklodowska-Curie grant agreement No 654857 and AK is supported by internal funds at Jagiellonian University No K/ZDS/006367.

## Acknowledgements

We would like to thank all students, researchers, clinicians, and educators who gave feedback in interviews, discussions, or the survey. We also thank Dr. Katja Kühlmeyer from the Institute for Ethics, History and Theory of Medicine at LMU Munich, who advised and supported us on the study design. Finally, we thank Prof. Leslie Fall, Prof. Martin Fischer, and Martin Adler for supporting the project.

## Competing interests

The authors declare that they have no competing interests. 

## Erratum

The name of the second author Kononowicz was originally misspelled (Kononowic).

## Supplementary Material

Data resources on which the grounded theory is based on

Virtual patient related data resources

Summary of the findings of step 2 - the application of the framework to virtual patients - for each category

## References

[1] Norman G (2005). Research in clinical reasoning: past history and current trends. Med Educ.

[2] Audétat MC, Dory V, Nendaz M, Vanpee D, Pestiaux D, Perron NJ, Charlin B (2012). What is so difficult about managing clinical reasoning difficulties?. Med Educ.

[3] Charlin B, Lubarsky S, Millette B, Crevier F, Audétat MC, Charbonneau A, Caire Fon A, Hoff L, Bourdy C (2012). Clinical reasoning processes: unravelling complexity through graphical representation. Med Educ.

[4] Ellaway RH, Poulton T, Smothers V, Greene P (2009). Virtual patients come of age. Med Teach.

[5] Ellaway R, Candler C, Greene P, Smothers V (2006). An Architectural Model for MedBiquitous Virtual Patients.

[6] Kononowicz AA, Zary N, Edelbring S, Corral J, Hege I (2015). Virtual patients-what are we talking about? A framework to classify the meanings of the term in healthcare education. BMC Med Educ.

[7] Cook D, Triola MM (2009). Virtual patients: a critical literature review and proposed next steps. Med Educ.

[8] Wiener F (1974). A system for the computer simulation of clinical reasoning. Comput Programs Biomed.

[9] Edelbring S, Broström O, Henriksson P, Vassiliou D, Spaak J, Dahlgren LO, Fors U, Zary N (2012). Integrating virtual patients into courses: follow-up seminars and perceived benefit. Med Educ.

[10] Berman NB, Fall LH, Smith S, Levine DA, Maloney CG, Potts M, Siegel B, Foster-Johnson L (2009). Integration Strategies for Using Virtual Patients in Clinical Clerkships. Acad Med.

[11] Courteille O, Bergin R, Stockeld D, Ponzer S, Fors U (2008). The use of a virtual patient case in an OSCE-based exam – A pilot study. Med Teach.

[12] Edelbring S (2013). Research into the use of virtual patients is moving forward by zooming out. Med Educ.

[13] Glaser BG, Holton J (2004). Remodeling Grounded Theory. Forum Qual Sozialforsch.

[14] Strauss A, Corbin J (1996). Grundlagen Qualitativer Sozialforschung.

[15] Barrows HS, Pickell GC (1991). Developing clinical problem-solving skills. A guide to more effective diagnosis and treatment.

[16] Watling CJ, Lingard L (2012). Grounded theory in medical education research: AMEE Guide No. 70. Med Teach.

[17] Eva K (2005). What every teacher needs to know about clinical reasoning. Med Educ.

[18] Durning S, Artino AR, Pangaro L, van der Vleuten CP, Schuwirt L (2011). Context and clinical reasoning: understanding the perspective of the expert's voice. Med Educ.

[19] Marcum JA (2013). The Role of Emotions in Clinical Reasoning and Decision Making. J Med Philos.

[20] De Figueiredo AD (2005). Learning Contexts: a Blueprint for Research. Inter Educ Multimedia.

[21] Durning S, Dong T, Artino AR, LaRochelle MJ, Pangaro LN, van der Vleuten C, Schuwirth L (2012). Instructional Authenticity and Clinical Reasoning in Undergraduate Medical Education: A 2-Year, Prospective, Randomized Trial. Mil Med.

[22] Patel R, Sandars J, Carr S (2015). Clinical diagnostic decision-making in real life contexts: A trans-theoretical approach for teaching: AMEE Guide No. 95. Med Teach.

[23] Rencic J (2011). Twelve tips for teaching expertise in clinical reasoning. Med Teach.

[24] Gunning WT, Fors UG (2012). Virtual Patients for assessment of medical student ability to integrate clinical and laboratory data to develop differential diagnoses: Comparison of results of exams with/without time constraints. Med Teach.

[25] Zimmerman B (1990). Self-Regulated Learning and Academic Achievement: An overview. Educ Psychol.

[26] Kassirer JP (2010). Teaching clinical reasoning: case-based and coached. Acad Med.

[27] Heitzmann N, Fischer F, Kühne-Eversmann L, Fischer MR (2015). Enhancing Diagnostic Competence with Self-Explanation Prompts andAdaptable Feedback. Med Educ.

[28] Lajoie SP (2005). Extending the scaffolding metaphor. Instr Sc.

[29] Bowen JL (2006). Educational Strategies to Promote Clinical Diagnostic Reasoning. N Engl J Med.

[30] Wallden S, Mäkinen E (2014). Educational Data Mining and Problem-Based Learning. Inform Educ.

[31] Tworek J, Coderre S, Wright B, McLaughlin K (2010). Virtual Patients - ED-2 Band-Aid or Valuable Asset in the Learning Portfolio?. Acad Med.

[32] Issenberg SB, McGaghie WC, Petrusa ER, Lee Gordon D, Scalese RJ (2005). Features and uses of high-fidelity medical simulations that lead to effective learning: a BEME systematic review. Med Teach.

[33] Cendan J, Lok B (2012). The use of virtual patients in medical school curricula. Adv Physiol Educ.

[34] Durning SJ, Dong T, Artino AR, van der Vleuten C, Holmboe E, Schuwirth L (2015). Dual processing theory and experts' reasoning: exploring thinking on national multiple-choice questions. Perspect Med Educ.

[35] Charlin B, Boshuizen HP, Custers EJ, Feltovich PJ (2007). Scripts and clinical reasoning. Med Educ.

[36] Berner ES, Graber ML (2008). Overconfidence as a cause of diagnostic error in medicine. Am J Med.

[37] Graber ML (2009). Educational strategies to reduce diagnostic error: can you teach this stuff?,. Adv Health Sci Educ Theory Pract.

[38] Bryce DA, King NJ, Graebner CF, Myers JH (1998). Evaluation of a Diagnostic Reasoning Program (DxR): Exploring Student Perceptions and Addressing Faculty Concerns. J Inter Media Educ.

[39] Durning SJ, Lubarsky S, Torre D, Dory V, Holmboe E (2015). Considering "Nonlinearity" Across the Continuum in Medical Education Assessment: Supporting Theory, Practice, and Future Research Directions. J Contin Educ Health Prof.

[40] van Bruggen L, van Woudenbergh M, Spierenburg E, Vos J (2012). Preferred question types for computer-based assessment of clinical reasoning: a literature study. Perspect Med Educ.

[41] Lubarsky S, Dory V, Duggan P, Gagnon R, Charlin B (2013). Script concordance testing: From theory to practice: AMEE Guide No. 75. Med Teach.

[42] Torre DM, Durning SJ, Daley BJ (2013). Twelve tips for teaching with concept maps in medical education. Med Teach.

[43] Wolpaw T, Papp KK, Bordage G (2009). Using SNAPPS to Facilitate the Expression of Clinical Reasoning and Uncertainties: A Randomized Comparison Group Trial. Acad Med.

[44] Round J, Conradi E, Poulton T (2009). Improving assessment with virtual patients. Med Teach.

[45] Kiesewetter J, Kager M, Lux R, Zwissler B, Fischer MR, Dietz I (2014). German undergraduate medical students' attitudes and needs regarding medical errors and patient safety - A national survey in Germany. Med Teach.

[46] Mamede S, Schmidt HG, Rikers R (2007). Diagnostic errors and reflective practice in medicine. J Eval Clin Pract.

[47] Posel N, McGee JB, Fleiszer DM (2015). Twelve tips to support the development of clinical reasoning skills using virtual patient cases. Med Teach.

[48] Smith S, Cookson J, McKendree J, Harden RM (2007). Patient-centred learning-back to the future. Med Teach.

[49] Botezatu M, Hult H, Fors UG (2010). Virtual patient simulation: what do students make of it? A focus group study. BMC Med Educ.

[50] Pinnock R, Spence F, Chung A, Booth R (2012). evPaeds: undergraduate clinical reasoning. Clin Teach.

[51] Bateman J, Allen M, Samani D, Kidd J, Davies D (2013). Virtual patient design: exploring what works and why. A grounded theory study. Med Educ.

[52] Fall LH, Berman NB, Smith S (2005). Whit CB, Woodhead JC, Olson AL. Multi-institutional Development and Utilization of a Computer-Assisted Learning Program for the Pediatrics Clerkship: The CLIPP Project. Acad Med.

[53] Huwendiek S, Reichert F, Bosse HM, de Leng BA, van der Vleuten CP, Haag M, Hoffmann GF, Tönshoff B (2009). Design principles for virtual patients: a focus group study among students. Med Educ.

[54] Rivera-Gutierrez DJ, Kopper R, Kleinsmith A, Cendan J, Finney G, Lok B (2014). Exploring Gender Biases with Virtual Patients for High Stakes Interpersonal Skills Training. Lect Note Comp Sci.

[55] Pataki C, Pato MT, Sugar J, Rizzo AS, Parsons TD, St (2012). George C, Kenny P. Virtual Patients as Novel Teaching Tools in Psychiatry. Acad Psych.

[56] Deladisma AM, Cohen, Stevens A, Wagner P, Lok B, Bernard T, Oxendine C, Schumacher L, Johnsen K, Dickersone R, Raij A, Wells R, Duerson M, Harper G, Lind S (2007). Do medical students respond empathetically to a virtual patient?. Am J Surg.

[57] Cook DA, Erwin PJ, Triola MM (2010). Computerized Virtual Patients in Health Professions Education: A Systematic Review and Meta-Analysis. Acad Med.

[58] Huwendiek S, Duncker C, Reichert F, De Leng BA, Dolmans D, van der Vleuten CP, Haag M, Hoffmann GF, Tönshoff B (2013). Learner preferences regarding integrating, sequencing and aligning virtual patients with other activities in the undergraduate medical curriculum: A focus group study. Med Teach.

[59] Kim S, Phillips WR, Pinsky L, Brock D, Phillips K, Keary J (2006). A conceptual framework for developing teaching cases: a review and synthesis of the literature across disciplines. Med Educ.

[60] Forsberg E, Ziegert K, Hult H, Fors U (2014). Clinical reasoning in nursing, a think-aloud study using virtual patients - A base for an innovative assessment. Nurse Educ Today.

[61] Kernt M, Holzer M, Bauer D, Fischer MR (2008). Concept Mapping zur Unterstützung der differentialdiagnostischen Hypothesenbildung im fallbasierten Online-Lernsystem CASUS: Qualitative Verbesserung der Diagnosefindung durch ICD-10 Kodierung. GMS Z Med Ausbild.

[62] Schladen MM (2015). Formative Research on Instructional Design Theory for Virtual Patients in Clinical Education: A Pressure Ulcer Prevention Clinical Reasoning case. Doctoral Dissertation 2015.

[63] Friedman CP, France CL, Drossman DD (1991). A Randomized Comparison of Alternative Formats for Clinical Simulations. Med Decis Making.

[64] Talbot TB, Sagae K, John B, Rizzo AA (2012). Sorting out the virtual patient: How to exploit artificial intelligence, game technology and sound educational practices to create engaging role-playing simulations. Intern J Gaming Comp Media Sim.

[65] Voelker R (2003). Virtual Patients Help Medical Students Link Basic Science With Clinical Care. JAMA.

[66] Nirenburg S, McShane M, Beale S (201). Aspects of Metacognitive Self-Awareness in Maryland Virtual Patient.

