# Supplementary material for: Advancing clinical reasoning in virtual patients – development and application of a conceptual framework
Source: GMS J Med Educ. 2018 Feb 15;35(1):Doc12. doi: 10.3205/zma001159 (PMC5827186; doi:10.3205/zma001159)
Supplement: Data resources on which the grounded theory is based on [file JME-35-12-s-001.pdf]

Literature, websites, and videos
Aagaard E, Teherani A, Irby DM. Effectiveness of the one-minute preceptor model for diagnosing the patient and the learner: Proof of concept. Acad Med 2004;79: 42-9.
Akturk AO, Sahin I. Literature Review on Metacognition and its Measurement.Procedia Social and Behavioral Sciences 2011; 15:3731-6.
All AC, Huycke LI. Serial concept maps: tools for concept analysis. J Nurs Educ 2007;46 (5):217-24.
Artino AR Jr. Think, feel, act: motivational and emotional influences on military students' online academic success. J Comput High Educ 2009; 21:146–166.
Artino AR Jr, Holmboe ES, Durning SJ. Control-value theory: Using achievement emotions to improve understanding of motivation, learning, and performance in medical education: AMEE Guide No. 64. Med Teach 2012; 34: e148–e160.
Artino AR, Cleary TJ, Dong T, Hemmer PA, Durning SJ. Exploring clinical reasoning in novices: a self-regulated learning microanalytic assessment approach. Med Educ 2014;48:280-91.
Audétat MC, Dory V, Nendaz M, Vanpee D, Pestiaux D, Junod Perron N, Charlin B. What is so difficult about managing clinical reasoning difficulties? Med Educ 2012; 46: 216–27.
Barrows HS, Pickell GC. Developing clinical problem-solving skills. A guide to more effective diagnosis and treatment. Norton Medical Books, W W Norton & Company Inc, New York, London 1991. ISBN 978-0-3937-1010-6
Ber R. The CIP (comprehensive integrative puzzle) assessment method. Med Teach 2003;25(2):171-6.
Bereiter C, Scardamalia M. Expertlike Learner in Surpassing Ourselves: An Inquiry into the Nature and Implications of Expertise. Chicago: Open Court, 1993, ISBN 0-8126-9204-7; 279.
Berner ES, Graber ML. Overconfidence as a cause of diagnostic error in medicine. Am J Med. 2008;121(5 suppl):S2–S23.
Beullens J, Struyf E, Van Damme B. Do extended matching multiple-choice questions measure clinical reasoning? Med Educ 39:410–417.
Bordage G. Prototypes and semantic qualifiers: From past to present. Med Educ 2007;41:1117-21.
Bowen JL. Educational Strategies to Promote Clinical Diagnostic Reasoning. N Engl J Med 2006;355:2217-25.
van Bruggen L, Manrique-van Woudenbergh M, Spierenburg E, Vos J. Preferred question types for computer-based assessment of clinical reasoning: a literature study. Perspect Med Educ 2012; 1:162-171.
de Bruin AB, Schmidt HG, Rikers RM. The Role of Basic Science Knowledge and Clinical Knowledge in Diagnostic Reasoning: A Structural Equation Modeling Approach. Acad Med 2005;80(8):765-73.
Cary J, Kurtz S. Integrating clinical communication with clinical reasoning and the broader medical curriculum. Patient Education and Counseling 2013;92: 361-5.
Case SM, Swanson DB. Constructing Written Test Questions For the Basic and Clinical Sciences. National Board of Medical Examiners Philadelphia, PA. http://www.uclouvain.be/cps/ucl/doc/adef/documents/EVA_Res_Ext_Questions_QCM.pdf
Chamberland M, Mamede S, St-Onge C, Setrakian J, Bergeron L, Schmidt H. Self-explanation in learning clinical reasoning: the added value of examples and prompts. Med Educ 2015; 49(2):193-202.

Charlin B, Lubarsky S, Millette B, Crevier F, Audétat MC, Charbonneau A, Caire Fon N, Hoff L, Bourdy C. Clinical reasoning processes: unravelling complexity through graphical representation. Med Educ 2012;46: 454-63

Charlin B, Boshuizen HP, Custers EJ, Feltovich PJ. Scripts and clinical reasoning. Med Educ 2007;41(12):1178-84.

CMap Tool http://cmap.ihmc.us/cmaptools/

Cox R. Vicarious learning and case-based teaching: developing health science students' clinical reasoning skills. Teaching and Learning Research Programme 54 2008.

Croskerry, Singhal G, Mamede S. Cognitive debiasing 1: origins of bias and theory of debiasing. BMJ Qual Saf 2013;22:ii58–ii64.

Daley BJ, Torre DM. Concept maps in medical education: an analytical literature review. Med Educ. 2010;44(5):440-8.

Delany C, Golding C. Teaching clinical reasoning by making thinking visible: an action research project with allied health clinical educators. BMC Med Educ 2014;14:20.

Downes S. What is learning context? 2004. http://www.downes.ca/post/18

Durning S, Artino AR Jr, Pangaro L, van der Vleuten CP, Schuwirth L. Context and clinical reasoning: understanding the perspective of the expert's voice. Med Educ 2011;45: 927-38.

Durning SJ, Artino A, Boulet J, et al. The feasibility, reliability, and validity of a post-encounter form for evaluating clinical reasoning. Med Teach 2012;34:30–37.

Durning S, Dong T, Artino AR Jr, LaRochelle MJ, Pangaro LN, van der Vleuten C, Schuwirth L. Instructional Authenticity and Clinical Reasoning in Undergraduate Medical Education: A 2-Year, Prospective, Randomized Trial. Mil Med 2012;177(9): 38

Durning SJ, Lubarsky S, Torre D, Dory V, Holmboe E. Considering "Nonlinearity" Across the Continuum in Medical Education Assessment: Supporting Theory, Practice, and Future Research Directions. J Contin Educ Health Prof 2015;35(3): 232-43.

Durning S, Ting D, Artino AR, van der Vleuten C, Holmboe E, Schuwirth L. Dual processing theory and experts' reasoning: exploring thinking on national multiple-choice questions. Perspect Med Educ 2015; 4:168–175.

Elizondo-Omana RE, Morales-Gómez JA, Morquecho-Espinoza O, Hinojosa-Amaya JM, Villarreal-Silva EE, García-Rodríguez Mde L, Guzmán-López S. Teaching Skills to Promote Clinical Reasoning in Early Basic Science Courses. Anat Sci Educ 2010;3:267–271

Ely JW, Graber ML, Croskerry P. Checklists to Reduce Diagnostic Errors. Acad Med. 2011;86:307–13.

Epp CM, Bull S, Johnson MD. Visualising Uncertainty for Open Learner Model Users. 22nd Conference on User Modelling, Adaptation and Personalization (UMAP 2014), Aalborg, Denmark. 9-14

Ericsson KA. Deliberate practice and the acquisition and maintenance of expert performance in medicine and related domains. Acad Med 2004;79(10 suppl):S70-80.

Eva K. What every teacher needs to know about clinical reasoning. Med Educ 2005; 39: 98-106.

Ferreira AP, Ferreira RF, Rajgor D, Shah J, Menezes A, Pietrobon R. Clinical Reasoning in the Real World Is Mediated by Bounded Rationality: Implications for Diagnostic Clinical Practice Guideline. PLoS ONE 2010;5(4) e10265.

de Figueiredo AD. Learning Contexts: a Blueprint for Research. Interactive Educational Multimedia 2005; 11:127-39.

Fischer MR. Presentation at Dartmouth: Clinical Reasoning: A special case of Scientific Reasoning and Argumentation? Oct. 2014. http://media.dartmouth.edu/~screencasts/d1250j1/Leading_Voices_in_Medical_Education_-_Clinical_Rea_-_20141107_153657_6.html

Fleming A, Cutrer W, Reimschisel T, Gigante J. You Too Can Teach Clinical Reasoning! Pediatrics 2012;130(5): 795-7.

Gauthier G, Lajoie SP. Do expert clinical teachers have a shared understanding of what constitutes a competent reasoning performance in case-based teaching? Instr Sci 2014; 42:579–94.

Graber ML. Educational strategies to reduce diagnostic error: can you teach this stuff? Adv in Health Sci Educ Theory Pract 2009; 14(Suppl 1): 63-9.

Greller W, Drachsler H. Translating Learning into Numbers: A Generic Framework for Learning Analytics. Educational Technology & Society 2012;15(3): 42-57.

Groothoff JW, Frenkel J, Tytgat GA, Vreede WB, Bosman DK, ten Cate OT. Growth of analytical thinking skills over time as measured with the MATCH test. Med Educ 2008: 42:1037-43.

Händel M, Artelt C, Weinert S. Assessing metacognitive knowledge: Development and evaluation of a test instrument. Journal for Educational Research Online, Journal für Bildungsforschung Online 2013; 5(2) 162-88.

Heitzmann N, Fischer F, Kühne-Eversmann L, Fischer MR. Enhancing Diagnostic Competence with Self-Explanation Prompts and Adaptable Feedback. Med Educ 2015;49(10):993-1003.

The writings of Hippocrates and Galen by Hippokrates, Galen, Coxe JR. Charles Rivers Editors (1846)

Horwitz LI, Moin T, Green ML. Development and Implementation of an Oral Sign-out Skills Curriculum. J Gen Intern Med. 2007;22(10): 1470-4.

Hrynchak P, Takahashi SG, Nayer M. Key-feature questions for assessment of clinical reasoning: a literature review. Med Educ 2014;48: 870–83.

iMedTrust. Adaptive Medical Education. http://www.imedtrust.org/adaptive-medical-education/

Irish College of General Practitioners. Modified Essays Questions (MEQ). http://www.icgp.ie/go/become_a_gp/micgp_examination/past_papers/modified_essay_question_meq_

Kamnin C, O'Sullivan P, Deterding R, Younger M. A Comparison of Critical Thinking in Groups of Third-year Medical Students in Text, Video, and Virtual PBL Case Modalities. Acad Med 2003; 78:204-11.

Kassirer JP. Teaching clinical reasoning: case-based and coached.Acad Med. 2010;85(7):1118-24.

Kiesewetter J, Kager M, Lux R, Zwissler B, Fischer MR, Dietz I. German undergraduate medical students' attitudes and needs regarding medical errors and patient safety - A national survey in Germany. Med Teach 2014;36: 505-10.

Kiesewetter J, Ebersbach R, Görlitz A, Holzer M, Fischer Mr, Schmidmaier R. Cognitive problem solving patterns of medical students correlate with success in diagnostic case solutions. PlosONE 2013;8(8):e71486.

Kinchin IM, Hay DB. How a qualitative approach to concept map analysis can be used to aid learning by illustrating patterns of conceptual development. Educ Res 2000;42 (1):43–57.

Lajoie SP. Extending the scaffolding metaphor. Instr Sc 2005;33:541-57.

Lee A, Joynt GM, Lee AK, Ho AM, Groves M, Vlantis AC, Ma RC, Fung CS, Aun CS. Using illness scripts to teach clinical reasoning skills to medical students. Fam Med 2010;42(4):255-61.

Leony D, Muñoz-Merino PJ, Ruipérez-Valiente JA, Martín-Caro DA, Kloos CD. Rule-based detection of emotions in the Khan Academy platform. In: International Workshop on Massive Open Online Courses, 15 -16 May 2014, Antigua, Guatemala.

Lubarsky S, Dory V, Duggan P, Gagnon R, Charlin B. Script concordance testing: From theory to practice: AMEE Guide No. 75. Med Teach 2013;35: 184-93.

Lucey C. From Problem List to Illness Scripts. http://c.ymcdn.com/sites/www.improvediagnosis.org/resource/resmgr/EducationFiles/From_Problem_Lists_to_Illnes.pdf

Mamede S, Schmidt HG, Rikers R. Diagnostic errors and reflective practice in medicine. J Eval Clin Pract 2007;13:138-45.

Mandin H, Harasym P, Eagle C, Watanabe M. Developing a 'clinical presentation' curriculum at the University of Calgary. Acad Med 1995;70:186–93.

Marcum JA. The Role of Emotions in Clinical Reasoning and Decision Making. Journal of Medicine and Philosophy 2013; 38: 501–19.

Medical Education Scholarship and Support UofS. Illness Script Examples. 2015. https://www.youtube.com/watch?v=MOUV8c9esuI&feature=youtu.be

Midgley C, Maehr ML, Hruda LZ, Anderman E, Anderman L, Freeman KE, Gheen M, Kaplan A, Kumar R, Middleton MJ, Nelson J, Roeser R, Urdan T. Manual for the Patterns of Adaptive Learning Scales. Ann Arbor, Mich: University of Michigan; 2000.

Moulton CA, Regehr G, Mylopoulos M, MacRae HM. Slowing down when you should: a new model of expert judgement. Acad Med 2007;82 (Suppl):109–16.

Nie Y, Li L, Duan Y, Chen P, Barraclough BH, Zhang M, Li J. Patient safety education for undergraduate medical students: A systematic review. BMC Med Educ 2011;11:33.

NMC Horizon Report 2013. http://net.educause.edu/ir/library/pdf/HR2013.pdf

Normak P, Pata K, Kaipainen M. An Ecological Approach to Learning Dynamics. Educational Technology & Society 2012;15 (3), 262–74.

Norman G. Research in clinical reasoning: past history and current trends. Med Educ 2005; 39:418-27

Page G, Bordage G, Allen T. Developing key-feature problems and examinations to assess clinical decision-making skills. Acad Med 1995;70(3):194-201.

Palmer EJ, Devitt PG. Assessment of higher order cognitive skills in undergraduate education: modified essay or multiple-choice questions? BMC Med Educ. 2007;7(49):1-7.

Patel R, Sandars J, Carr S. Clinical diagnostic decision-making in real life contexts: A trans-theoretical approach for teaching: AMEE Guide No. 95. Med Teach 2015;37(3):211-27.

Patient Safety Network. WebM&M Cases & Commentaries. https://psnet.ahrq.gov/webmm

Papp KK. Critical Thinking Behavior Scale (CTBS): A Validation Study.

Pitt MB, Borman-Shoap EC, Eppich WJ. Twelve tips for maximizing the effectiveness of game-based learning. Med Teach 2015;37:1013-7.

Power T. Strategic Thinking Concepts-SMART Tool. 2014. https://www.youtube.com/watch?v=oHIyqqOZAz0&feature=youtu.be

Pusic MV, Boutis K, Hatala R, Cook DA. Learning Curves in Health Professions Education. Acad Med 2015;90(8):1034-42.

Rencic J. Twelve tips for teaching expertise in clinical reasoning. Med Teach 2011;33: 887–892.

Retalis S, Papasalouros A, Psaromiligkos Y, Siscos S, Kargidis T. Towards Networked Learning Analytics - A concept and a tool. Networked Learning 2006.

Richards J. Critical Synthesis Package: Comprehensive Integrative Puzzle (CIP) . MedEdPORTAL Publications; 2013. https://www.mededportal.org/publication/9561

Ruipérez-Valiente JA, Muñoz-Merinoa PJ, Leonya D, Kloosa CD. ALAS-KA: A learning analytics extension for better understanding the learning process in the Khan Academy platform. Computers in Human Behavior 47 2015;139-48.

Schmidt HG, Rikers RM. How expertise develops in medicine: knowledge encapsulation and illness script formation. Med Educ 2007; 41: 1133-39.

Schmidt HG, Mamede S. How to improve the teaching of clinical reasoning: a narrative review and a proposal. Med Educ 2015;49(10):961-73.

School of Nursing and Midwifery, Faculty of Health, University of Newcastle. Clinical Reasoning Instructors Resources. http://www.utas.edu.au/__data/assets/pdf_file/0003/263487/Clinical-Reasoning-Instructor-Resources.pdf

Semigarn HL, Linder JA, Gidengil C, Mehrotra A. Evaluation of symptom checkers for self diagnosis and triage: audit study. BMJ 2015;351:h3480

Shackleton-Jones N. Towards a Working Theory of Learning: The Affective Context Model. 2010. http://www.aconventional.com/2010/05/towards-working-theory-of-learning.html

Shackleton-Jones N. Understanding the Affective Context Model. 2010. http://www.aconventional.com/2010/08/understanding-learning-affective.html

Sharples M, Adams A, Ferguson R, Gaved M, McAndrew P, Rienties B, Weller M, Whitelock D. Open University Innovation Report 3. 2014 http://www.openuniversity.edu/sites/www.openuniversity.edu/files/The_Open_University_Innovating_Pedagogy_2014_0.pdf

Shum SB, Ferguson R. Social Learning Analytics. Educational Technology & Society 2012;15(3):3-26.

Society to improve Diagnosis in Medicine. Clinical Reasoning Toolkit. http://www.improvediagnosis.org/?ClinicalEducation

Stiegler M. Understanding and Preventing Cognitive Errors in Healthcare. Stanford University 2014. https://www.youtube.com/watch?v=OXcGciywtgM&feature=youtu.be

Tempelaar DT, Rienties B, Giesbers B. In search for the most informative data for feedback generation: Learning analytics in a data-rich context. Computers in Human Behavior 47 2015; 157-67.

Torre DM, Durning SJ, Daley BJ. Twelve tips for teaching with concept maps in medical education. Med Teach 2013;35(3):201-8.

University of Conneticut. Teacher-centered vs Learner-centered paradigms. http://assessment.uconn.edu/docs/TeacherCenteredVsLearnerCenteredParadigms.pdf

Verbert K, Manouselis N, Drachsler H, Duval E. Dataset-Driven Research to Support Learning and Knowledge Analytics. Educational Technology & Society 2012;15(3):133-48.

Vink SC, van Tartwijk J,Bolk J, Verloop N. Integration of clinical and basic sciences in concept maps: a mixed-method study on teacher learning. BMC Medical Education 2015; 15:20

Wallden S, Mäkinen E. Educational Data Mining and Problem-Based Learning. Informatics in Education 2014;13(1):141-56.

West DC, Park JK, Pomeroy JR, Sandoval J. Concept mapping assessment in medical education: a comparison of two scoring systems. Med Educ 2002;36:820–6.

WHO Guidelines on a Patient Safety Curriculum. http://www.who.int/patientsafety/education/curriculum/download/en/

WHO Learning from error video and booklet. http://www.who.int/patientsafety/education/vincristine_download/en/

Wikipedia. Self-regulated learning. https://en.wikipedia.org/wiki/Self-regulated_learning

Wikipedia. Shared Decision Making. https://en.wikipedia.org/wiki/Shared_decision-making

Williams PA. A six-item mnemonic to reduce cognitive errors during medical diagnosis. Poster session presented at: Diagnostic error in medicine. 3rd International conference of the Society for Medical Decision Making, 2010 October 25–27. Toronto, Canada.

Wolpaw T, Papp KK, Bordage G. Using SNAPPS to Facilitate the Expression of Clinical Reasoning and Uncertainties: A Randomized Comparison Group Trial. Acad Med 2009; 84(4): 517-23.

Woods NN. Science is fundamental: the role of biomedical knowledge in clinical reasoning. Med Educ 2007;41: 1173-77.

Zimmerman B. Self-Regulated Learning and Academic Achievement: An overview. Educational Psychologist 1990; 25(1):3-17

Interviews

Interview partner	Topic
Two healthcare educators	Implementation of a clinical reasoning course for undergraduate medical students
Healthcare educator	Team reasoning
Healthcare education researcher	Learning from errors
Computer scientist	Concept mapping to visualize clinical reasoning
Healthcare professional	Clinical reasoning and management decisions

Online courses

University of Technology Delft. Creative Problem Solving and Decision Making. MOOC 2015. https://courses.edx.org/courses/course-v1:DelftX+TPM1x+2T2015/info

University of Michigan. Instructional Methods in Health Professions Education. MOOC 2015. https://www.coursera.org/course/instructmethodshpe
University of California San Francisco. Clinical Problem Solving. MOOC 2015. https://www.coursera.org/course/clinprobsolv
