# Supplementary material for: Advancing clinical reasoning in virtual patients – development and application of a conceptual framework
Source: GMS J Med Educ. 2018 Feb 15;35(1):Doc12. doi: 10.3205/zma001159 (PMC5827186; doi:10.3205/zma001159)
Supplement: Virtual patient related data resources [file JME-35-12-s-002.pdf]

Literature, websites, videos

AAMC Institute for Improving Medical Education. Effective Use of Educational Technology in Medical Education Colloquium on Educational Technology: Recommendations and Guidelines for Medical Educators. 2007. Available from https://members.aamc.org/eweb/upload/Effective%20Use%20of%20Educational.pdf

Adler M. Entwicklung einer differentialdiagnostischen Netzwerk-Komponente für die medizinische multimediale Datenbank CASUS. Dissertation 1996. Technische Universität München.

Bateman J, Allen M, Samani D, Kidd J, Davies D. Virtual patient design: exploring what works and why. A grounded theory study. Med Educ 2013;47: 595–606.

Botezatu M, Hult H, Fors UG. Virtual patient simulation: what do students make of it? A focus group study. BMC Medical Education 2010; 10:91

Bryce DA, King NJ, Graebner CF, Myers JH. Evaluation of a Diagnostic Reasoning Program (DxR): Exploring Student Perceptions and Addressing Faculty Concerns. Journal of Interactive Media in Education, 1998;98 (1):1-35. Available from http://www-jime.open.ac.uk/articles/10.5334/1998-1/

Cook DA, Erwin PJ, Triola MM. Computerized Virtual Patients in Health Professions Education: A Systematic Review and Meta-Analysis. Acad Med 2010;85:1589–1602.

Cook DA, Triola MM. Virtual patients: a critical literature review and proposed next steps. Med Educ 2009;43: 303-11.

Deladisma AM. Cohen, Stevens A, Wagner P, Lok B, Bernard T, Oxendine C, Schumacher L, Johnsen K, Dickersone R, Raij A, Wells R, Duerson M, Harper G, Lind S. Do medical students respond empathetically to a virtual patient? The American Journal of Surgery 2007;193: 756–60.

Fall LH, Berman NB, Smith S. Whit CB, Woodhead JC, Olson AL. Multi-institutional Development and Utilization of a Computer-Assisted Learning Program for the Pediatrics Clerkship: The CLIPP Project. Acad Med 2005; 80(9): 847-54.

Forsberg E, Ziegert K, Hult H, Fors U. Clinical reasoning in nursing, a think-aloud study using virtual patients - A base for an innovative assessment. Nurse Educ Today 2014;34(4):538-42.

Friedman CP, France CL, Drossman DD. A Randomized Comparison of Alternative Formats for Clinical Simulations. Med Decis Making 1991;11: 265.

Goal-based Scenarios. 2012. http://teorije-ucenja.zesoi.fer.hr/doku.php?id=instructional_design:goal_based_scenarios

Gunning WT, Fors UG. Virtual Patients for assessment of medical student ability to integrate clinical and laboratory data to develop differential diagnoses: Comparison of results of exams with/without time constraints. Med Teach 2012;34: e222–e228.

Helyen D, Nijholt A, op den Akker R. Affect in tutoring dialogues. Applied Artificial Intelligence 2005;19:287–311.

Huwendiek S, Duncker C, Reichert F, De Leng BA, Dolmans D, van der Vleuten CP, Haag M, Hoffmann GF, Tönshoff B. Learner preferences regarding integrating, sequencing and aligning virtual patients with other activities in the undergraduate medical curriculum: A focus group study. Med Teach 2013; 35: 920–929

Huwendiek S, Reichert F, Bosse HM, de Leng BA, van der Vleuten CP, Haag M, Hoffmann GF, Tönshoff B. Design principles for virtual patients: a focus group study among students Med Educ 2009;43(6):580-8.

Issenberg SB, McGaghie WC, Petrusa ER, Lee Gordon D, Scalese RJ. Features and uses of high-fidelity medical simulations that lead to effective learning: a BEME systematic review. Med Teach 2005; 27(1):10–28.

Kernt M, Holzer M, Bauer D, Fischer MR. Concept mapping for supporting the differential diagnostic generation of hypotheses in the case-based online learning system CASUS: Qualitative improvement of dagnostic performance through ICD-10 coding. GMS Zeitschrift für Medizinische Ausbildung 2008, Vol. 25(3).

Kim S, Phillips WR, Pinsky L, Brock D, Phillips K, Keary J. A conceptual framework for developing teaching cases: a review and synthesis of the literature across disciplines. Med Educ 2006; 40(9):867-76

MedBiquitous Virtual Patient Standard. http://www.medbiq.org/working_groups/virtual_patient/index.html

Nirenburg S, McShane M, Beale S. Aspects of Metacognitive Self-Awareness in Maryland Virtual Patient. Cognitive and Metacognitive Educational Systems: Papers from the AAAI Fall Symposium (FS-10-01), 2010; 69-74.

Pataki C, Pato MT, Sugar J, Rizzo AS, Parsons TD, St. George C, Kenny P. Virtual Patients as Novel Teaching Tools in Psychiatry. Acad Psych 2012;36(5): 398-400.

Pinnock R, Spence F, Chung A, Booth R. evPaeds: undergraduate clinical reasoning. Clin Teach 2012;9(3):152-7.

Posel N, McGee JB, Fleiszer D. Twelve tips to support the development of clinical reasoning skills using virtual patient cases. Med Teach 2015;37(9):813-8

Rivera-Gutierrez DJ, Kopper R, Kleinsmith A, Cendan J, Finney G, Lok B. Exploring Gender Biases with Virtual Patients for High Stakes Interpersonal Skills Training. Lecture Notes in Computer Science 2014;8637:385–396.

Round J, Conradi E, Poulton T. Improving assessment with virtual patients. Med Teach 2009;31: 759–763.

Salminen H, Zary N, Björklund K, Toth-Pal E, Leanderson C. Virtual Patients in Primary Care: Developing a Reusable Model That Fosters Reflective Practice and Clinical Reasoning. J Med Internet Res 16(1): e3

Sanders C, Kleinert HL, Boyd SE, Herren C, Theiss L, Mink J. Virtual patient instruction for dental students: can it improve dental care access for persons with special needs? Spec Care Dentist 2008;28(5):205-13

Schladen MM. Formative Research on Instructional Design Theory for Virtual Patients in Clinical Education: A Pressure Ulcer Prevention Clinical Reasoning case. Doctoral Dissertation 2015. Nova Southeastern University. http://nsuworks.nova.edu/gscis_etd/35/

Smith S, Cookson J, McKendree J, Harden RM. Patient-centred learning-back to the future. Med Teach 2007; 29: 33–37.

Smith S, Kogan JR, Berman NB, Dell MS, Brock DM, Robins LS. The Development and Preliminary Validation of a Rubric to Assess Medical Students' Written Summary Statements in Virtual Patient Case. Acad Med 2016; 91(1):94-100
Talbot TB, Sagae K, John B, Rizzo AA. Sorting out the virtual patient: How to exploit artificial intelligence, game technology and sound educational practices to create engaging role-playing simulations. International Journal of Gaming and Computer-Mediated Simulations. 2012;4(3):1-19. doi: 10.4018/jgcms.2012070101
Tworek J, Coderre S, Wright B, McLaughlin K.Virtual Patients: ED-2 Band-Aid or Valuable Asset in the Learning Portfolio? Acad Med 2010;85(1): 155-8.
Voelker R. Virtual Patients Help Medical Students Link Basic Science With Clinical Care. JAMA, 290(13):1700-1.
Waldman Z, Ottolini M. Differential Diagnosis: Approaches and Pitfalls - A Pediatric Case-Based Session for 3rd Year Medical Students. MedEdPORTAL Publications; 2013. Available from: https://www.mededportal.org/publication/9640
Wilson J. Evaluating the Effectiveness of Virtual Patients to Promote Clinical Reasoning. Dissertation Northcentral University 2011. http://gradworks.umi.com/34/62/3462968.html

**Virtual patients, authoring guidelines and systems**
Overview of virtual patient systems. http://vpsystems.virtualpatients.net
Campus. http://www.medizinische-fakultaet-hd.uni-heidelberg.de/CAMPUS-Software.109992.0.html, https://virtuellepatienten.de/eviphd/index.html?guest=true&vp=hp_lumbalpunktion
CaseManager. https://vetmedcases.osu.edu/, http://odee.osu.edu/sites/default/files/2012b_vetmed_finalreport_final.pdf
CaseTrain. https://casetrain.uni-wuerzburg.de/index.shtml
CASUS. http://player.casus.net
DecisionSim and VPSim. https://www.kynectiv.com/experience, http://vpsim.pitt.edu/shell/CaseList_Assignments.aspx
DxR. http://www.dxrgroup.com/products/clinician/
evPaeds. https://flexiblelearning.auckland.ac.nz/evpaeds_example/1.html
i-Human. http://www.i-human.com/media-room/videos-b-roll/
Inmedea Simulator. http://www.inmedea-simulator.net
OpenLabyrinth. http://openlabyrinth.ca/, http://openlabyrinth.ca/user-guide/
SBL Interactive. http://scenarios.sblinteractive.org/Scenario/List
ShadowHealth http://shadowhealth.com/health-assessment.html
Vic. http://pie.med.utoronto.ca/vic/VIC_content/VIC_familyMedicine.html
Virtual Surgical Patient. https://www.discoursellc.com/, https://www.youtube.com/watch?v=RK0kiD9EMVI&feature=youtu.be
