# Supplementary material for: Advancing clinical reasoning in virtual patients – development and application of a conceptual framework
Source: GMS J Med Educ. 2018 Feb 15;35(1):Doc12. doi: 10.3205/zma001159 (PMC5827186; doi:10.3205/zma001159)
Supplement: Summary of the findings of step 2 - the application of the framework to virtual patients - for each category [file JME-35-12-s-003.pdf]

Concept	Findings
Context (learning and clinical context)	
Time	• Time constraints for learning [e.g. 24, 49] vs. encouraging learner to pause and reflect [e.g. 50]<br>• Typically time is a static component in VPs, learners are either rewarded for spending a long time on the VP [e.g. CASUS [54]] or for a quick problem solving approach, in which case a timer may be present [e.g. OpenLabyrinth [http://vpsystems.virtualpatients.net]
Authenticity, cognitive load	• The design of VP influences the balance between authenticity & complexity [e.g. 51]<br>• Cognitive load of VPs is related to errors [e.g. 51]<br>• VPs offer a controlled/safe environment in which teachers can focus on the learner [e.g. 32]<br>• How does the transfer from virtual to real world work? [e.g. 31]<br>• VPs are often based on fictive patient stories [e.g. 52], but are often implemented to prepare for real patient encounters [e.g. 53]
Emotions	• Emotions of the **patient** are more common in VPs to train communication skills than in clinical reasoning VP formats [e.g. 54, 55]<br>• Emotions of the **learner** during interaction with VP are explored [e.g. 51, 56]
Learner-centeredness	
Feedback	• Feedback is an important determinant for engagement [e.g. 57] and varies from immediate feedback to feedback at the end of a scenario.<br>• Different/adaptable feedback mechanisms have been implemented [e.g. 51, 31], but typically it is quantitative (e.g. list of correct diagnoses) [VP systems [http://vpsystems.virtualpatients.net]]
Adaptability	• VPs are typically tailored to the needs and level of a specific learner group, not on an individual level [http://vpsystems.virtualpatients.net], but<br>• Adaption of level of difficulty helps avoiding an expertise reversal effect [e.g. 7] and the<br>• Optimal design may depend on learner level [e.g. 7], meaning offering multiple learning strategies in varying context [e.g. 32, 47]<br>• Overall, there is a discrepancy between adaptability and standardization of instruction [e.g. https://members.aamc.org/eweb/upload/Effective%20Use%20of%20Educational.pdf]]
Motivation, Engagement	• Motivation through assessment [e.g. 58]<br>• Importance of making a VP engaging [e.g. 59]
Teaching/Assessment	
Methods	• VPs are assessment methods [e.g. 11], but also include assessment components, which are typically quantitative methods, such as multiple choice questions or decision points. [VP systems [http://vpsystems.virtualpatients.net]]<br>• Applying more qualitative methods, such think-aloud approaches [e.g. 60] or concept mapping have been studied [e.g. 61], but are not commonly used.<br>• Many cases are required to assess clinical reasoning skills [e.g. 7]<br>• Mimic real patient management vs reliability [e.g. 44]
Scoring	• Assess and score all steps involved in the clinical reasoning process, but a detailed scoring of a stepwise approach may oppose non-analytical reasoning approaches [e.g. 7]<br>• Scoring can cause negative emotions, such as frustration, thus, transparency of scoring is important [e.g. 51, 62]<br>• The scoring approach depends on the VP format [e.g. 63]<br>• Scoring metrics vary, e.g. costs or time are included in some VPs [e.g. 47], but, specific clinical reasoning process scores are often not provided [VP systems [http://vpsystems.virtualpatients.net]]
Communication	• Clear communication is an indicator for organized clinical reasoning skills [e.g. 47], but VPs are often designed to train either communication or clinical reasoning skills [e.g. 64]
Patient-centeredness [e.g. 48]	
Patient Safety & Errors	• VPs provide a safe environment for learners and educators [e.g. https://members.aamc.org/eweb/upload/Effective%20Use%20of%20Educational.pdf] and errors can be made, by making a wrong choice or giving a wrong answer, however, typical errors/biases of clinical decision making, such a anchoring or premature closure typically cannot actively made by the learner [VP systems [http://vpsystems.virtualpatients.net]]<br>• Learning from errors is important [e.g. 47], but implementation is limited.

Biases	• Biases are usually not explicitly covered in clinical reasoning VPs, but studies have been implemented [e.g. 54] exploring the role of biases while learning with VPs.
Management decisions	• Management decision are usually included in the VP scenario before the conclusion of the scenario [VP systems [http://vpsystems.virtualpatients.net]]
Overconfidence	• Level of confidence with a differential or final diagnosis is implemented in some VPs [e.g. DxR [http://vpsystems.virtualpatients.net]]
Psychological Theories	
Illness Scripts	• Patient's illness scripts are implicitly included in VPs, but typically not explicitly visible to the learner and<br>• the learner typically has to enter (or is provided) a list of problems, differential diagnoses, tests to perform, and management decisions [VP systems [52]
Knowledge Encapsulation	• Often, VPs are long [e.g. 52], which hinders working through many VPs<br>• Longitudinal integration of VPs, to link basic and clinical knowledge [e.g. 65]
Metacognition	• Role of metacognitive self-awareness of the VP [e.g. 66]<br>• Metacognitive strategies, such as questions for self-reflection, self-assessment or self-explanation are implemented in VPs [VP systems [http://vpsystems.virtualpatients.net]<br>• Self-pacing, non-threatening environment, feedback and flexibility important requirements for metacognition [e.g. 38]
Pattern recognition - analytical reasoning	• VPs typically aim to teach analytical reasoning in a step-wise approach [e.g. 49]
